# Sensory Profile of Rice-Based Snack (Nuroongji) Prepared from Rice with Different Levels of Milling Degree

**DOI:** 10.3390/foods9060685

**Published:** 2020-05-26

**Authors:** Mina K. Kim

**Affiliations:** Department of Food Science and Human Nutrition and Fermented Food Research Center, Jeonbuk National University, 567 Baekjedaero, Deokjin-gu, Jeonju-si, Jeonbuk 54896, Korea; minakim@jbnu.ac.kr; Tel.: +82-63-270-3879

**Keywords:** Nuroongji, rice snack, descriptive analysis, consumer acceptance testing, consumer studies, texture, mouthfeel

## Abstract

Nuroongji is a traditional rice-based snack that is widely consumed in Korea, but there is no reported comprehensive sensory characterization of this popular snack. The objective of this study was to conduct a sensory analysis of Nuroongji made with rice with different degrees of milling. Four different types of Nuroongji samples according to the degree of milling were prepared in the lab and subjected to physiochemical analysis. Descriptive sensory analysis was conducted by a trained panel (*n* = 8), and a consumer acceptance test was conducted using college students (*n* = 70). A sensory lexicon describing the flavor and texture characteristics of Nuroongji was developed: it included roasted brown rice, burnt, buckwheat, rice powder, glutinous rice power, and floral. The following texture attributes were evaluated in triplicate: hardness of particles, irregularity of particles, degree of coagulation, number of chews, and residual mouthfeel. Significant differences in flavor and mouthfeel attributes were observed between the Nuroongji samples according to the degree of milling (*p* < 0.05). Nuroongji made with white rice (N1) had a higher hardness value and less sweetness compared to other samples (*p* < 0.05). Texture- and mouthfeel-related attributes such as cohesiveness of the mass, irregularity of the surface, and astringency were identified as important characteristics that drive consumer acceptance of Nuroongji products. Findings from this study can provide Nuroongji product developers a valuable insight to extend their market by reformulating the product to be appealing to young consumers.

## 1. Introduction

The market for gluten-free products is increasing as consumer awareness toward health and wellness increases. Approximately 1%–2% of the population in Western countries is suffering from celiac disease [1] and this equates to about 2.1 million adults in the U.S. [2]. While gluten-free product choice is essential only for those who suffer from celiac disease, health-conscious consumers are also choosing gluten-free products as healthier alternatives for their regular wheat-based products [3]. This indicates that food choice behavior among consumers is affected by not only sensory but also non-sensory factors including psychological factors [4], context of food consumption [5], emotional affects [6], and sustainability [7]. Sensory and non-sensory factors influence consumer food choice behavior and understanding factors affecting the food choice is crucial for market success. Due to the rise of the gluten-free market, products with gluten-free options are well studied, these include, prebiotic gluten-free bread [1] and gluten-free bread with different functional food additives [8,9] or lactic acid cultures [10]. These gluten-free options of wheat-based products do not completely mimic the sensory characteristics of their standard counterpart products. Studies reported that gluten-free version of wheat-based food products are more costly [11,12] and several products were contaminated with gluten in gluten-free products [13]. While there are limitations on gluten-free alternatives of standard wheat-based products, processed rice products are naturally non-gluten containing and can be served as gluten-free products in nature. 

Nuroongji is a traditional rice-based snack that is widely consumed in Korea. It is typically had as an accompaniment to tea or soup [14]. Nuroongji is the scorched rice that is left at the bottom of the cooker [15]. Traditionally, this scorched rice was scratched off the bottom of the cooker and eaten as a snack. The traditional method of making Nuroongji is highly dependent on what is left at the bottom of the rice cooker; therefore, the sensory quality and/or quantity of traditionally made Nuroongji is not well controlled. Recently, companies have started to mass-produce Nuroongji, and the typical method of commercially preparing it is as follows: Rice is washed, soaked in water for 30–40 min, and cooked. The cooked rice is pressed in a heated device that resembles a waffle-maker. The cooked rice is roasted and dried as a result of the heated pressing device, and this is how the brown and crispy characteristics of Nuroongji are achieved [16]. This process of browning and crisping can be explained by the Maillard browning reaction [15]. There have been extensive sensory studies on cooked rice, but very little research has been conducted on the sensory characteristics of Nuroongji. The summary of previously reported studies on Nuroongji can be found in Table 1. 

These mentioned studies primarily focused on products with little additions to Nuroongji; therefore, sensory characterization was not well-considered. While previous study on Nuroongji only focused on quality characterization, many studies were conducted on sensory characterization of cooked rice with differing milling degrees. Previous study reported the effect of milling ratio on sensory characteristics of cooked rice, and documented 4 appearance-related terms (color, glossiness, intactness of grains, plumpness); 9 flavor attributes including boiled egg white, puffed corn, dairy, raw rice, wet cardboard, hay-like, metallic, sweet, and bitter tastes; and 10 texture-related attributes including degree of agglomeration, adhesiveness, roughness, hardness, cohesiveness, inner moisture, cohesiveness of mass, chewiness, toothpacking, and residuals [23]. Their study reported that degree of agglomeration, adhesiveness, inner moisture, toothpacking, and cohesiveness of mass of cooked rice increased with increased milling degree. Their work compared the instrumental texture analysis and sensory texture analysis data to comprehensively provide the texture profiles of cooked rice with different milling degrees. As Nuroongji is also a rice-based product, Nuroongji and cooked rice may share similar sensory characteristics, while there may also be inevitable differences in the sensory characteristics as a result of the additional processing. To our knowledge, sensory characterization of Nuroongji is lacking. Understanding sensory characteristics of Nuroongji can provide valuable insights to those who are seeking a healthier snack as well as those who are on gluten-free diets. Therefore, the objective of this study was to identify the sensory characteristics of Nuroongji made with rice subjected to different degrees of milling. Descriptive sensory analyses using a well-trained sensory panel and consumer acceptance testing were carried out in order to identify the sensory characteristics that drove consumer liking of Nuroongji. 

## 2. Materials and Methods 

### 2.1. Preparation of Nuroongji Samples 

Rice (*Oryza sativa* subsp. *japonica* cultivar Koshihikari) harvested in Gimje, Jeollabukdo, was purchased directly from a supplier. This supplier does not mill the rice upon harvesting, and the stored brown rice is milled according to the requirement of the customer. Brown rice, white rice, 50% milled rice, and 70% milled rice were prepared by the supplier on order and were shipped to the lab immediately after milling. The different rice samples were refrigerated to minimize the possibility of the formation of an oxidized note. The Nuroongji samples were prepared in the lab using a Nuroongji maker (BE-7200; Bethelcook, Hwasung, Korea). In order to make Nuroongji, the four rice samples were washed three times with tap water and soaked in water for 40 min at 20 °C. Then, the rice was cooked for 40 min at 110 °C using a rice cooker (PERW-20L, PN; Ansan, Gyeonggi-do, Korea) following the manufacturer’s recommended method of cooking. Upon cooking, 42.5 g of cooked rice (1.5 oz) was scooped out using an ice cream scooper (#20, Minyong Corp. Jeonju, Korea) and was immediately transferred to the Nuroongji maker (BE-7200, Bethel Electronics, Hwaseong-si, Gyeonggi-do, Korea), in which it was pressed for 4 min. The pressed rice removed from the Nuroongji maker is the commercially available crispy snack (6.5 cm × 12 cm × 0.2 cm) that is referred to as Nuroongji. Nuroongji made with unmilled brown rice was labeled as N1; Nuroongji made with 50% milled rice was labeled as N2; Nuroongji made with 70% milled rice was labeled as N3; and Nuroongji made with white rice was labeled as N4. A total of 20 Nuroongji snacks (6.5 cm × 12 cm × 0.2 cm) were prepared for physiochemical analysis, and 200 Nuroongji snacks were prepared for sensory analysis in order to minimize the batch-to-batch variability. 

Once the Nuroongji samples were prepared, they were additionally cooked for further analysis. Based on a comprehensive review of cook books, online recipes, and other sources of information, the following cooking method was used: 25 g of crushed Nuroongji was added to 500 mL of cold water in a pot (5% w/v) and boiled for 10 min with the lid on. Immediately after cooking, the samples were kept in a warming cabinet set at a temperature of 60 °C for further analysis.

### 2.2. Physiochemical Analysis of Nuroongji Samples 

Physiochemical analysis of pH, moisture content, color, and water-binding capacity was conducted using standard methods of analysis. Briefly, the pH of the Nuroongji samples was measured with a pH meter (Lab 850; Schott, Germany) after filtering 10% (w/v, with distilled water) diluted Nuroongji through a filter paper (Whatman No. 2; Maidstone, UK). Moisture content was measured using a moisture analyzer (WBA-110M; Daihan, Korea), and color was analyzed using a color analyzer (CR-10; Minolta, Osaka, Japan). A color value standard plate was used with values L* = 97.51, a* = −5.26, and b* = +7.00. For moisture content analysis, the Nuroongji sample was crushed to a powder (that could pass through a mesh 30) and tightly packed in a clear container designated for the color analyzer, and L*, a*, b* values of the Nuroongji samples were measured. The water-binding capacity of Nuroongji was assessed using the method from previous study [15]. Briefly, the Nuroongji sample was crushed to a powder (that could pass through a mesh 30), and 0.5 g of the powder was added to 30 mL of distilled water. It was then mixed thoroughly with a magnetic stirrer for 1 h at room temperature. Once thoroughly mixed, this mixture was centrifuged at 3000 × *g* for 30 min, and the supernatant was removed. The weight of the solid residue was measured, and the ratio of the solid residue to the powder was considered as the water-binding capacity (%). All analyses were conducted in triplicate. 

### 2.3. Descriptive Sensory Analysis 

In the descriptive sensory analysis, the flavor and texture (mouthfeel) components of each of the four Nuroongji samples were evaluated by a well-trained panel of eight members (two males and six females aged 22–40 years). Each panelist was recruited as part of a taster club in Jeonbuk National University in 2016 and were screened based on their interests and ability of basic taste recognition and odor recognition. Panelists were initially trained for 80 h in the Spectrum^TM^ method. After initial training, panelists have been participating the sensory evaluation of various food products. Prior to the evaluation, each panelist received 40 h of training with a basic taste solution for aroma recognition, aroma expression, and intensity rating with the universal scale used in the Spectrum^TM^ method. In addition to training, eight 2 h training sessions were held to generate a sensory lexicon for flavor and texture as well as a method for evaluating the textural attributes of the Nuroongji samples. Separate sensory lexicons were generated for flavor and texture with various reference samples including food and chemical references. For the flavor lexicon, references were provided for each attribute in order to minimize the panelist-to-panelist variation resulting from aroma recognition and expression. Texture attributes were evaluated following previous texture evaluation protocols for rice with some modifications [24]. Briefly, panelists evaluated the texture attributes via a three-stage approach: In stage 1, they evaluated the “hardness” and “irregularity” of the Nuroongji sample by pressing the sample against their tongue. In stage 2, another spoonful (~6 g) of the sample was placed in the mouth in order to evaluate the “cohesiveness” of the particles while chewing. Finally, in stage 3, another spoonful (~6 g) of the Nuroongji sample was placed in the mouth, in order to evaluate the “number of chews” before swallowing (or spitting) and “residual mouthfeel” upon swallowing (or spitting). 

The Nuroongji samples were cooked using the method mentioned earlier: 25 g of the Nuroongji sample was added to 500 mL of cold water in a pot (5% w/v) and boiled for 10 min with the lid on. Different batches of Nuroongji were crushed and mixed thoroughly before cooking in order to minimize the batch-to-batch variation. Immediately after cooking, 50 mL of the samples was poured into a 195 mL paper cup (Jin-kwang Papers; Paju, Gyeonggi-do, Korea) that was labeled with a three-digit random code and covered with a lid, and kept in the warming cabinet set at 60 °C. All samples were prepared 30 min prior to evaluation, and the samples were served and evaluated at 60 °C. Panelists were asked to evaluate the flavor attributes in triplicate, and separate sessions were held for texture evaluation. 

For texture evaluation, the panelists were asked to place a spoonful of the sample and follow the three-stage approach described above. All the samples were evaluated in triplicate by each panelist (*n* = 6) with a randomized, balanced design. A 2 min rest was enforced between samples in order to minimize the carry-over effect in the flavor evaluation session. The panelists recorded their intensity ratings on a paper ballot, and scored on the 15-point universal scale of the Spectrum^TM^ method. Upon completion of the flavor and texture evaluation, the panelists were invited to an appreciation dinner as compensation for their participation in the analysis. 

### 2.4. Consumer Acceptance Testing 

For consumer acceptance testing of the four Nuroongji samples, a total of 70 consumers participated. Participants were recruited from Jeonbuk National University—Jeonju campus through flyers, SNS postings, and personal contacts. The majority of the participants were active members of the Food Taster Club operated by Jeonbuk National University—Sensory Service Center, and hence, they were undergraduate students. Participants in this study did not represent the Korean population, as majority of participants were college students. Drawing a conclusion based on the consumer acceptance testing data from this study may pose a risk of potential bias. Although the potential bias of participant sampling exists, participants were screened to ensure that they were either users of Nuroongji products (consume such products at least one a month) or at least a product acceptor. The Nuroongji samples were prepared using the same protocol used in the descriptive sensory analysis. In order to keep the consistency of cooked Nuroongji, participants were pre-scheduled for their evaluation time. All samples were prepared 30 min prior to consumer testing, and the samples were served in a paper cup with the lid on and labeled with a three-digit random code (as described for the descriptive sensory analysis). Samples were served monadically, and the order of presentation was randomized and balanced.

On the day of testing, participants were asked to fill out a brief demographic questionnaire prior to evaluation, and then asked to visually evaluate the appearance and color of the Nuroongji samples. The participants were asked to rate their responses to the following attributes: appearance and color likings; overall liking; and other likings such as flavor, texture, spreadability, and stickiness. Their responses were scored on a nine-point hedonic scale with 1 indicating dislike extremely, 5 indicating neither dislike nor like, and 9 indicating like extremely, as suggested by previous study [25]. 

### 2.5. Statistical Analysis 

Results were expressed as mean values from triplicate analyses of the physiochemical properties of the samples. Mean values of flavor and texture from the triplicate evaluations by each panelist (*n* = 8) were reported for the descriptive sensory analysis, and the mean liking scores collected from the 70 consumer test participants were reported. Analysis of variance followed by Fisher’s least significant difference (for the physiochemical analysis) and Tukey’s honestly significant difference (for the descriptive sensory analysis and the consumer acceptance test) was conducted at the α = 0.05 level. Partial least square regression was conducted with the overall liking score as a single Y-axis variable and the physiochemical attributes and sensory flavor and texture attributes as X-axis variables, under the assumption that each X variable had an equal chance of influencing the model. All statistical analyses were conducted using XLSTAT (v.2019; Addinsoft, Paris, France). 

## 3. Results and Discussion

### 3.1. Physiochemical Analysis 

The physiochemical analysis results for the Nuroongji samples can be found in Table 2. Significant differences in physiochemical properties were observed between the Nuroongji samples (*p* < 0.05). Briefly, the pH of the Nuroongji samples ranged from 6.32 to 6.61, with N1 having the highest pH value and N3 having the lowest value (*p* < 0.05). The moisture content of the Nuroongji samples exhibited significant differences: N2 and N4 had a significantly higher moisture content than N1 and N3 (N2 = 9.07%, N4 = 8.39%, N3 = 5.93%, N1 = 5.54%). It must be noted that all the samples were prepared with the same method and the same machine and were prepared within a period of 1 week, so any differences in moisture content were unlikely to be associated with the preparation method. The water-binding capacity also showed significant differences: N3 and N4 had the highest water-binding capacities, while N1 had the lowest water-binding capacity (253.3%) (*p* < 0.05). The low moisture content (5.93%) of N3 may have influenced its high water-binding capacity (486.7%). Previously, it was reported that the water-binding capacity of Nuroongji was inversely correlated with the protein content of rice [20]. Additionally, the protein content of brown rice is higher than that of white rice and rice with different degrees of milling [20]. This might explain the low water-binding capacity of N1, as N1 was unmilled brown rice. 

The color of the Nuroongji samples also showed significant differences (*p* < 0.05). Before cooking, the L* value of the samples ranged from 57.50 to 71.47; the a* value ranged from 16.30 to 24.3; and the b* value ranged from 6.70 to 15.90. These values were in agreement with previously reported values [17,26,27]. After cooking, the L* of the Nuroongji samples ranged from 6.70 to 15.90; the a* value ranged from 2.43 to 4.30; and the b* value ranged from 5.90 to 9.13. Before cooking, the L* value of N3 was the lowest; this indicates that it was darker in color (*p* < 0.05). The b* value of N1 was the highest at 24.3, which is indicative of a yellowish color. N1 was made with brown rice; therefore, the yellow color could be attributed to the rice germ that was remaining in the brown rice. 

### 3.2. Descriptive Sensory Analysis 

The descriptive sensory analysis results for the four Nuroongji samples can be found in Table 3 (flavor attributes) and Table 4 (texture attributes). A total of 12 flavor attributes, including 6 aromas, 5 basic tastes, and 1 mouthfeel attribute, were utilized to describe the flavor characteristics. Five texture-related attributes, including hardness, irregularity, cohesiveness, numbers of chews, and residual mouthfeel, were utilized to describe the texture characteristics. 

The Nuroongji samples made with unmilled brown rice (N1) and 70% milled rice (N3) shared similar flavor characteristics: that is, the aroma intensities for roasted brown rice and buckwheat tea were significantly higher than those of the other samples (*p* < 0.05), while the aroma intensities for rice powder and sticky rice powder were significantly lower than those of the other samples (*p* < 0.05). No differences were observed between N1 and N3 with regard to these four attributes (*p* > 0.05). When the aroma characteristics of N1 (Nuroongji made with brown rice) and N4 (Nuroongji made with white rice) were compared, the aroma intensity of roasted brown rice was significantly higher for N1 than for N4. A previous study on the attributes of seolgitteok, which is a type of Korean rice cake made by steaming rice flour, also reported higher levels of brown rice flavor in seolgitteok made with brown rice than in seolgitteok made with white rice [26]. The aroma intensities for rice powder and sticky rice powder were significantly higher in N4 than in the other samples (*p* < 0.05). Similarly, a previous study reported that the cooked rice flavor was higher in seolgitteok made with only white rice than in seolgitteok made with the addition of brown rice [26]. In the present study, a flowery aroma was noted in all the samples, and this flowery aroma has also previously been reported as an aroma attribute of cooked rice [27]. No significant differences were observed in basic tastes, except for sweet taste (*p* > 0.05): the intensity of sweet taste was the highest (1.99) for N4, which was followed by N2 (1.66), N3 (1.52), and N1 (1.10), which had the lowest sweet taste intensity (*p* < 0.05). In agreement with these findings, a previous study on descriptive analysis of cooked rice with differing degrees of milling also reported that cooked white rice had a more intense sweet taste than cooked brown rice [17,28]. 

With regard to the texture attributes, similar attributes that are used to describe cooked rice were utilized to describe the present Nuroongji samples (Table 4). For example, as mentioned earlier, the three-stage approach for evaluating cooked rice that was proposed by previous study [17] was utilized: the degree of agglomeration, adhesiveness, roughness, hardness, cohesiveness, and inner moisture was assessed in stage 1; the cohesiveness of the mass and chewiness were assessed at stage 2; and toothpacking and residuals were evaluated at stage 3. Additionally, three other texture-related terms, that is, hardness, number of chews (defined as “chewiness” in the previous study), and cohesiveness and residual mouthfeel (residuals) were similar to previously reported texture attributes for cooked rice [23]. Significant differences were observed between the present samples with regard to hardness, irregularity, number of chews, and residual mouthfeel (*p* < 0.0001). N1 had the highest hardness value, and the difference was significant compared to the other samples (*p* < 0.0001). Accordingly, a previous study also reported that brown rice had a higher hardness value than white rice [23,28]. 

### 3.3. Consumer Acceptance Testing

The results of the consumer acceptance test can be found in Table 5. A total of 70 consumers participated, and all of them were either users of Nuroongji products or were open to using them. Significant differences were observed in all the liking attributes (*p* < 0.05). Briefly, the overall liking scores of N2 and N3 were similar (*p* > 0.05) at 6.2 and 6.0, respectively. However, the overall liking score of N1 was significantly lower than that of N2 and N3, and N4 had the lowest overall liking score at 4.6 (*p* < 0.0001). Significant differences were observed in appearance-related attributes: N4 had the lowest appearance liking score (4.3) and color liking score (4.1), and the difference in the scores were significant in comparison with those for N1, N2, and N3 (*p* < 0.0001). In agreement with these findings, a previous study on the appearance and aroma characteristics of raw rice reported that highly milled rice was rated as being glossier than non- and/or lightly milled rice or brown rice after cooking [27]. Additionally, it has been reported that consumers preferred yellow instant Nuroongji to instant Nuroongji of a white color [17]. Similar to the trends for appearance and color liking, N4 received the lowest flavor liking score (4.6), and the difference was significant in comparison with those for N1, N2, and N3 (*p* < 0.0001). N2 remained at the top of the list with regard to the flavor liking score (6.6). With regard to the texture-related attributes texture, spreadability, and stickiness, N1 received the lowest liking scores of 4.9, 4.3, and 4.8, respectively. These scores were significantly lower than those of the other samples for all three attributes (*p* < 0.0001). This is as expected because previous study reported that consumers preferred Nuroongji with low hardness value when it is served in a soup type (5%–10% w/v in hot water) [20]. 

### 3.4. Physiochemical and Sensory Attributes Driving Consumer Likings

**Figure 1:**
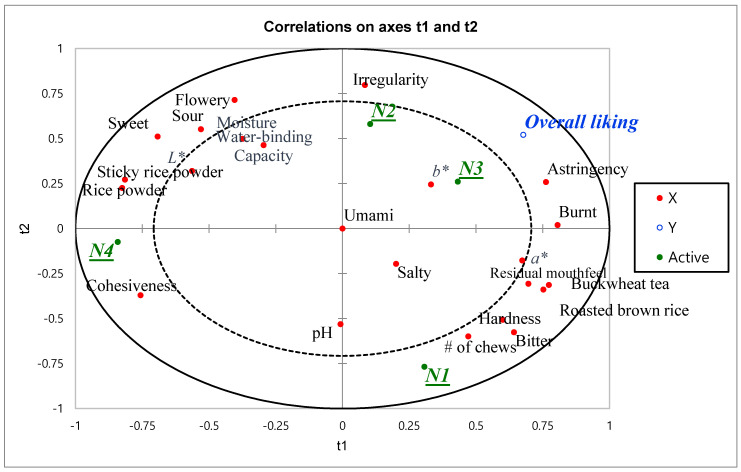
Partial least square regression on Nuroongji samples prepared with rice with different milling degree. N1 represents the Nuroongji made with unmilled brown rice; N2 represents Nuroongji made with 50% milled rice; N3 represents Nuroongji made with 70% milled rice; N4 represents Nuroongji made with white rice.

Partial least square regression analysis of the physiochemical analysis data, the sensory data obtained from the trained panel, and the overall consumer liking scores (Figure 1) showed that overall liking is best predicted by astringency and burnt aroma. Previous research on instant Nuroongji products has reported that a high degree of hardness showed the most significant correlation to consumer preference followed by yellow color of the instant Nuroongji sample [17]. However, the present findings did not clearly show a high correlation between overall liking and hardness or stickiness of Nuroongji products. Instead of flavor-related attributes, cohesiveness of the mass, irregularity of particles, and one mouthfeel attribute (astringency) were identified as important variables for consumer preference of Nuroongji. This is an interesting finding, because flavor remains the driver of liking for a majority of food products such as orange juice [29], chocolate milk [30], and Doenjang [31]. In contrast, the findings from this study indicated that texture and mouthfeel-related attributes, rather than flavor-related attributes, are important for consumers to determine their preference for Nuroongji products. This is also in agreement with the findings of previous studies on consumer preference for Nuroongji [16].

Texture attributes being drivers of consumer liking were previously documented in food commodities, especially the foods that offer healthier alternatives. Consumers preferred Greek yogurt with firm texture due to the increased protein content associated with firm yogurt [32]. Another study reported texture attributes such as creaminess, smoothness, cohesiveness, and mouth coating were important for consumer liking of spreadable cheese with varying fat contents [33]. Carbonated mouthfeel in lemon lime carbonated beverages was identified as a driver of liking among regular and diet beverage consumers. Softness of bread was also identified as a driver of liking for prebiotic gluten-free bread [1]. As seen in previous reports on drivers of liking of various food products, texture attributes became more important for consumers who seek healthier alternatives such as increased protein content [32], reduced fat [33], diet beverages [34], and gluten-free bread [1]. In the case of Nuroongji, which can be served as a gluten-free snack, texture attributes such as cohesiveness of the mass, irregularity of particles, and astringency were also identified as important variables for consumer liking. It is important to note that consumers who are seeking a healthier alternative do not want to sacrifice the sensory characteristics of the counterpart [35]. However, a product with healthier options (-free, - reduced, and -fortified) does not always created the same sensory characteristics in terms of flavor and texture, and this is a challenge in the food industry. Nuroongji, however, is naturally healthier snack in comparison to wheat-based snacks; therefore, there is no need for modification of the formulation for health-conscious consumers. Findings from this study suggest that texture-related attributes such as cohesiveness of the mass, irregularity of particles, and astringency are the key variables for consumer preference of Nuroongji, and this information can be useful for reformulating the Nuroongji to target consumers seeking gluten-free foods.

## 4. Conclusions

Differences in flavor and mouthfeel attributes were observed in Nuroongji products according to the degree of milling, and the differences were observed to a greater degree in the texture attributes. Importantly, the texture- and mouthfeel-related attributes cohesiveness of the mass, irregularity of the surface, and astringency were identified as important characteristics that drive consumer acceptance of Nuroongji products. Thus, texture, rather than flavor, seems to have a greater influence on consumer preference for Nuroongji products. Findings from this study can provide valuable information for market extension of Nuroongji toward consumers seeking gluten-free options. As stated earlier, food choice behavior is affected by various factors including sensory and non-sensory attributes. This study reported the sensory characteristics that drive consumer likings of Nuroongji using college students. The limitation of this study was the use of college students for consumer acceptance testing. Nuroongji is a traditional snack/tea in Korea, and the primary consumers of Nuroongji are older populations. In order to completely understand the consumer behavior, additional consumer testing using different consumer segments such as older populations and/or those who are actually on gluten-free diets is necessary in order to define the sensory drivers of liking of Nuroongji among the Korean population. This part will be done in a future study. In addition to sensory factors affecting Nuroongji, defining non-sensory factors affecting consumer purchase behavior of Nuroongji is another area of future study. Overcoming the “traditional” and “old” image of Nuroongji is another challenge for market extension. More research has to be done focused only on Nuroongji in order to expand the market share, and market expansion of Nuroongji can eventually promote the rice consumption in Korea, as Nuroongji is a processed rice product.

## Figures and Tables

**Table 1:** Summary of previous research on Nuroongji products.

Authors	Subject Studied	Key Finding
Suh et al. (1996) [16]	Cooking conditions of Nuroongji	Nuroongji prepared with cabinet cooker showed higher consumer preference
Park and Oh (1997) [17]	Instant Nuroongji	Physiochemical characteristics of instant Nuroongji made with steam cooker, pressure cooker, and cabinet cooker were documented
Cha (1999) [18]	Processing method for Nuroongji	Processing conditions such as hydrolysis with α-amylase and gelatinization of rice powder for Nuroongji preparation were studied
Lee et al. (2009a) [19]	Instant Nuroongji with added *Dioscorea japonica* powder	Quality characteristics of Nuroongji made with varying levels of *Dioscorea japonica* powder (2%, 4%, 6%, 8%) were profiled
Lee et al. (2009b) [20]	Instant Nuroongji prepared using a microwave	Quality characteristics of instant Nuroongji prepared by microwave, steam cooker, and pressure cooker were compared
Do et al. (2010) [14]	Nuroongji processing facilities	High microbial contamination in the process where employee interaction was required
Ha et al. (2012) [20]	Nuroongji made from *japonica* rice cultivars	Quality and sensory characteristics of Nuroongji made with different types of rice cultivars (Hopum, Shindongjin, Daeribbyeo, Chinnong, Boramchan, Deuraechan)
Yang and Choi (2016) [15]	Five commercial Nuroongji products	Antioxidant properties and physiochemical properties including pH, moisture, color were profiled
Choi et al. (2017) [21]	Pan bread with Nuroongji powder	Quality characteristics of pan bread with different levels of Nuroongji powder (5%, 10%, 15%, 20%) were studied
Lee (2018) [22]	Nuroongji with green whole grain	Quality characteristics of Nuroongji made with green whole grain was profiled

**Table 2:** Physiochemical properties of Nuroongji samples.

	pH	Moisture (%)	Water-Binding Capacity (%)	Before Cooking			After Cooking		
				L*	a*	b*	L*	a*	b*
N1	6.61 a	5.54 b	253.3 c	67.97 a	6.70 a	24.3 a	6.70 b	3.60 b	5.97 b
N2	6.50 ab	9.07 a	346.7 bc	68.13 a	3.83 b	16.30 b	9.40 b	2.60 c	5.90 b
N3	6.32 c	5.93 b	486.7 a	57.50 b	7.30 a	19.63 ab	13.33 a	4.30 a	9.13 a
N4	6.45 b	8.39 a	460.0 ab	71.47 a	3.80 b	17.13 b	15.90 a	2.43 c	6.37 b

Numbers represent the mean values of triplicate analyses of each physiochemical attributes; means in a column that do not share the same alphabet represent significant differences at the α = 0.05 level; N1 represents the Nuroongji made with unmilled brown rice; N2 represents Nuroongji made with 50% milled rice; N3 represents Nuroongji made with 70% milled rice; N4 represents Nuroongji made with white rice.

**Table 3:** Descriptive sensory flavor analysis results on Nuroongji samples.

Sensory Term	Definition	N1	N2	N3	N4	*p*-Value
Roasted brown rice	Typical aromatics associated with brown rice puff (Ref: roasted brown rice DuriDuri from Hansum Healthy Food Cooperative, Nonsan, Chungnam, Korea)	2.6 a	1.3 b	2.6 a	0.8 c	<0.0001
Burnt	Typical aromatics associated with burnt wood (Ref.: burnt match)	1.1 b	0.8 b	1.6 a	0.2 c	<0.0001
Buckwheat tea	Typical aromatics associated with buckwheat tea (Ref: buckwheat tea from Dongseo, Siheung, Gyeonggi-do, Korea)	2.2 a	1.6 b	2.2 a	1.3 b	<0.0001
Rice powder	Typical aromatics associated with rice powder (Ref: organic rice powder from Mom’s rice, Gwangju, Gyeonggi-do, Korea)	1.0 c	1.7 b	1.0 c	2.4 a	<0.0001
Sticky rice powder	Typical aromatics associated with sticky rice powder (Ref: organic sticky rice powder from Mom’s rice, Gwangju, Gyeonggi-do, Korea)	0.5 c	1.3 b	0.6 c	2.0 a	<0.0001
Flowery	Typical aromatics associated with chamomile tea (Ref: chamomile tea from e-mart, Seoul, Korea)	0.7 c	1.8 a	1.1 bc	1.5 ab	<0.0001
Sweet	Fundamental taste sensation of which sucrose is typical (sweet 2 = 1% (w/v) sucrose solution, sweet 5 = 5% (w/v) sucrose solution)	1.1 c	1.7 b	1.5 b	2.0 a	<0.0001
Sour	Fundamental taste sensation of which acetic acid is typical (sour 2 = 0.05% (w/v)sucrose solution, sour 5 = 0.08%(w/v) sucrose solution)	0.3 a	0.4 a	0.5 a	0.5 a	0.127
Salty	Fundamental taste sensation of which sodium chloride is typical (salty 2 = 0.36% (w/v) salt solution, salty 5 = 0.5% (w/v) salt solution)	0.4 a	0.3 a	0.5 a	0.4 a	0.793
Bitter	Fundamental taste sensation of which caffeine is typical (bitter 2 = 0.05% (w/v) caffeine solution, bitter 5 = 0.08%(w/v) caffeine solution)	0.6 a	0.3 a	0.4 a	0.2 a	0.750
Umami	Fundamental taste sensation of which monosodium glutamate is typical (umami 3 = 5% (w/v) monosodium glutamate solution)	0.6 a	0.6 a	0.6 a	0.6 a	0.980
Astringency	Drying mouthfeel on a tongue that is associated with tannins (0.1% tannic acid)	1.8 b	2.4 a	1.9 b	0.2 c	<0.0001

Numbers represent the mean values of triplicate analyses of each sensory term; means in a row that do not share the same alphabet represent significant differences at the α = 0.05 level; N1 represents the Nuroongji made with unmilled brown rice; N2 represents Nuroongji made with 50% milled rice; N3 represents Nuroongji made with 70% milled rice; N4 represents Nuroongji made with white rice.

**Table 4:** Descriptive sensory texture analysis results on Nuroongji samples.

Sensory Term		Definition	N1 ^(6)^	N2	N3	N4	*p*-Value ^(5)^
Stage 1 ^(1)^	Hardness	Force required to break the rice particles with tongue	11.4 ^(4)^ a	6.8 b	5.9 b	3.1 c	<0.0001
	Irregularity	Degree of irregularity of particle breakdown pattern	2.1 c	5.2 ab	5.9 a	4.1 b	<0.0001
Stage 2 ^(2)^	Cohesiveness	Degree of which samples sticks together in a mass while chewing	5.5 a	5.2 a	5.3 a	6.0 a	0.989
Stage 3 ^(3)^	# of chews	Numbers of chews required before spit (or swallow)	58.7 a	42.7 b	38.3 bc	34.3 c	<0.0001
	Residual mouthfeel	Amount of residual particles left in the mouth after spit (or swallow)	5.1 a	4.4 ab	4.0 b	3.0 c	<0.0001

^(1)^ Place a spoonful of Nuroongji sample (~6 g) in mouth and press the particles with tongue; ^(2)^ place a spoonful of Nuroongji sample (~6 g) in mouth and evaluate while chewing; ^(3)^ place a spoonful of Nuroongji sample (~6 g) and evaluate during and after swallowing; ^(4)^ numbers represent the mean values of triplicate analyses of each sensory term; ^(5)^ means in a row that do not share the same alphabet represent significant differences at the α = 0.05 level; ^(6)^ N1 represents the Nuroongji made with unmilled brown rice; N2 represents Nuroongji made with 50% milled rice; N3 represents Nuroongji made with 70% milled rice; N4 represents Nuroongji made with white rice.

**Table 5:** Consumer acceptance test results for Nuroongji samples.

	N1	N2	N3	N4	*p*-Value
Appearance liking	6.0 a	6.1 a	5.6 a	4.3 b	<0.0001
Color liking	6.2 ab	6.3 a	5.6 b	4.1 c	<0.0001
Overall liking	5.3 b	6.2 a	6.0 a	4.6 c	<0.0001
Flavor liking	6.0 ab	6.6 a	5.4 b	4.7 c	<0.0001
Texture liking	4.9 b	5.9 a	5.8 a	5.6 a	<0.001
Spreadability liking	4.3 b	5.4 a	5.9 a	5.6 a	<0.0001
Stickiness liking	4.8 b	5.5 a	5.9 a	5.4 a	<0.0001

Numbers in a row that does not share same alphabet represent significant differences at the α = 0.05 level. N1 represents the Nuroongji made with unmilled brown rice; N2 represents Nuroongji made with 50% milled rice; N3 represents Nuroongji made with 70% milled rice; N4 represents Nuroongji made with white rice.
